# In vitro evaluation of the percutaneous absorption of progesterone in anhydrous permeation-enhancing base using the Franz skin finite dose model and mass spectrometry

**DOI:** 10.1007/s00403-024-03040-x

**Published:** 2024-05-30

**Authors:** Daniel Banov, Guiyun Song, Kendice Ip, Erin H. Seeley, Stefan T. Linehan, Isabel Bassani, Gianna Ferron, August S. Bassani, Benigno C. Valdez

**Affiliations:** 1 Professional Compounding Centers of America (PCCA), 9901 South Wilcrest Drive, Houston, TX 77099 USA; 2 Department of Chemistry, University of Texas at Austin, Austin, TX 78712 USA; 3 SEP Scientific, Medfield, MA 02052 USA; 4 Department of Biology, Honors Program, Baylor University, Waco, TX 76798 USA; 5 TissueVision Inc, Newton, MA 02458 USA; 6 Department of Stem Cell Transplantation and Cellular Therapy, The University of Texas MD Anderson Cancer Center, Houston, TX 77030 USA

**Keywords:** Anhydrous permeation-enhancing base, Hormone replacement therapy, Progesterone

## Abstract

Progesterone is used for hormone replacement therapy through various routes of administration. This study was conducted to (a) evaluate the stability of progesterone in a proprietary anhydrous permeation-enhancing base (APEB) and the efficiency of its skin permeation, and (b) determine the appropriateness of mass spectrometry as a method of analysis for permeated progesterone. Using a proven stability-indicating ultra-performance liquid chromatographic method, the compounded hormone (100 mg progesterone/g APEB gel) was determined to be physically and chemically stable at room temperature for six months. Skin permeation analysis using the Franz skin finite dose model and mass spectrometry imaging showed an optical density of 1699 for the permeated progesterone compounded in APEB and 550 for the permeated progesterone in a water containing VBC, which is a statistically significant different (*P* = 0.029). The study suggests that APEB can be used as a compounding base for effective skin permeation of progesterone, and mass spectrometry is a reliable method for visualization and quantitative analysis of permeated progesterone.

## Introduction

Progesterone is a steroid sex hormone that plays an important role in menstruation and early pregnancy. It thickens the uterine lining that creates an appropriate environment for fertilized egg implantation. Lower levels of progesterone cause the uterine lining to break down and commences the menstrual cycle.

The level of progesterone decreases during menopause and causes uncomfortable symptoms including hot flashes, night sweats, headaches, mood changes, depression, vaginal dryness, weight gain and discomfort during sex; hence, progesterone hormone therapy is commonly recommended [1]. Progesterone may be administered through various routes including oral, intranasal, transdermal, vaginal, rectal, intramuscular, subcutaneous, and intravenous injection. Its route of administration should be judiciously selected because it affects the pharmacokinetics and efficacy of progesterone. For example, the oral bioavailability of progesterone is less than 10% due to its poor gastrointestinal tract absorption and extensive first-pass metabolism by the liver [2, 3]. Vaginal administration is preferred because it provides local therapeutic actions, ease of use, rapid absorption, and higher bioavailability but side effects including irritation and bleeding have been observed [4, 5]. The convenience, safety and non-invasiveness of transdermal delivery make it more popular. However, it has low bioavailability due to skin barriers [6]. This drawback is overcome by compounding progesterone with an appropriate base that improves its solubility and skin permeability. A prior study determined that liquid crystalline nanoparticles facilitated permeation of progesterone up to 65% of applied dose which was 6-fold better than the aqueous suspension [7], suggesting that a non-aqueous topical base for use in compounding with lipophilic drugs, such as progesterone, could provide for better delivery.

Additionally, a previous study showed that percutaneous absorption of progesterone in an anhydrous permeation-enhancing gel base (APEB, also called PCCA VersaBase® Anhydrous HRT), was significantly higher than absorption of progesterone in a water-containing VersaBase® Cream (VBC) [8]. However, the amount of progesterone distributed in various skin layers is unknown. The present study evaluates the skin distribution of progesterone, after application of an extemporaneously compounded progesterone gel and cream (APEB and VBC bases, respectively), using the Franz skin finite dose model and mass spectrometry. The method qualitatively and quantitatively analyzes the distribution of progesterone in human skin samples.

## Materials and methods

### Compounded topical formulation

The compounded formulations used in this study contained progesterone USP (PCCA Special Micronized) in either APEB or VBC from Professional Compounding Centers of America (Houston, TX). The ingredient’s list for the APEB gel is as follows: cyclopentasiloxane, caprylyl methicone, PEG-16 macadamia glycerides, polysilicone-11, PEG-12 dimethicone/PPG-20 crosspolymer, 1,2-hexanediol, phosphatidylcholine, jojoba esters, isopropyl jojobate, jojoba alcohol, and tocopheryl acetate. In contrast, the ingredient’s list for the aqueous VBC is as follows: water, emulsifying wax NF, ethylhexyl stearate, cyclopentasiloxane, sorbitol, aloe barbadensis leaf juice, glycerin, tocopheryl acetate, lactobacillus ferment, citric acid, disodium EDTA, sodium levulinate, sodium anisate, and sodium benzoate.

### Evaluation of the stability of progesterone compounded in APEB

#### a) UPLC parameters

The stability of progesterone was determined using Ultra-Performance Liquid Chromatography (UPLC) to separate progesterone from its degradation products. The mobile phase consisted of water with 0.1% trifluoroacetic acid (A), acetonitrile with 0.1% trifluoroacetic acid (B), acetonitrile (C), and 50% methanol in water (D). A 2.1 mm x 100 mm Waters UPLC Cortecs C18+ column with 1.6-µm particle size (Waters Corp., Milford, MA) maintained at 25°C was used to separate molecules with a flow rate of 0.35 mL/min. Molecules were detected at a 243 nm wavelength. Samples (1 µL) were injected into the Aquity UPLC separation module equipped with a photodiode array detector (Waters Corp.). Data were acquired and analyzed using Empower version 3 (Waters Corp.).

#### b) Validation of UPLC

The UPLC method was validated in accordance with the ICH “Harmonised Tripartide Guideline: Validation of Analytical Procedures: Text and Methodology Q2(R1)” [9] and the USP General Chapter: <1225 > Validation of Compendial Procedures [10] for system suitability, linearity, accuracy, precision, robustness, solution stability, and specificity. This validation was performed to demonstrate that the analytical procedure for progesterone 100 mg/g in APEB gel was a stability-indicating method, accurate and precise for the assay of progesterone. The specificity was assessed by subjecting the compounded formulation (0.4 g) to heat (80°C for 3 days), acid hydrolysis (0.2 N HCl at 40^o^C for 3 days), base hydrolysis (0.2 N NaOH at 40^o^C for 3 days), and oxidation (6% H_2_O_2_ at 40^o^C for 3 days).

To prepare the stock solutions for UPLC analysis, a precise quantity of progesterone was dissolved in HPLC grade methanol. The stock solution was then diluted with methanol to various concentrations as standard solutions.

Sample concentrations with weight corrections were calculated as previously described [11] and shown below.

Measured concentration:

$${c_x} = \left( {\left( {{A_r} - b} \right)/m} \right) \cdot {w_x}/{w_1} \cdot a \cdot [1 - l] \cdot ({d_x} \cdot {v_x})/{w_2}$$

where *c*_*x*_ = measured concentration of progesterone in sample.

*w*_*x* =_ target weight of progesterone topical gel in APEB sample.

*w*_*1*_ = actual weight of progesterone topical gel in APEB sample.

*a* = assay of progesterone standard.

*l =* loss of drying content in progesterone standard.

*d*_*x*_ = specific gravity of solvent.

*v*_*x*_ = volume of second dilution sample.

*w*_*2*_ = actual weight of second dilution sample.

#### c) Chemical stability of progesterone in solution

The stability of progesterone standard and sample solution in HPLC vials was evaluated for 1 day at 10 °C. Fresh solutions were prepared on day 0 and analyzed against a freshly prepared working standard solution according to the test methods. The solutions were re-analyzed against a freshly prepared working standard solution according to the test method at each time interval.

#### d) Stability of progesterone in APEB

A Beyond-Use Date (BUD) study for progesterone 100 mg/g APEB gel was performed using the validated sample preparation and instrumental conditions. All formulations were prepared and stored at room temperature (20^o^C – 25^o^C). The progesterone potency was analyzed after 14, 90, 120, 150 and 180 days. The recovery percentage of progesterone at the indicated time point was used to assess the stability of progesterone. The results from the stability study were reported as mean ± standard deviation from the two samplings. A retention of 90–110% of the initial concentration was considered stable.

### In vitro permeation test of progesterone compounded in APEB

#### a) Preparation of skin samples

Donated human cadaver abdomen skin tissues from one donor were purchased from BioIVT (Westbury, NY, USA). They were stored at − 20 °C in tightly sealed water-impermeable plastic bags until use. The skin samples were thawed at room temperature prior to use and soaked in a diffusion medium at room temperature for at least 30 min. The skin samples were then examined for signs of diseases and physical damage. To avoid skin cell death due to repeated freeze-thaw, no skin tissue was re-frozen.

#### b) Franz skin finite dose model

The skin samples were fitted into the Franz diffusion system (surface area of 1.77 cm^2^) as previously described [12]. Briefly, the recipient chamber of the Franz system was filled with 12 mL of a receptor solution (Phosphate-Buffered isotonic Saline (PBS), pH 7.4 ± 0.1 plus 0.5% of 2-hydroxypropyl-β-cyclodextrin and 50 µg/mL of gentamycin), and the chamber chimney left open to ambient laboratory conditions. All Franz diffusion chambers were connected to a circulating water bath and the skin surface temperature was maintained at 32.0 °C ± 1.0 °C. The receptor medium contained within each diffusion cell was mixed at approximately 600 RPM using a magnetic stirring device to ensure appropriate homogenization of the release drug in the receptor phase throughout the experiment.

A Precision LCR meter set at low voltage alternating current was used to determine the integrity of the skin samples. An electrical resistance of 4 kΩ was used as a cut-off value; any skin with resistance less than 4 kΩ was rejected [13] as this value corresponds to a tritiated water permeability coefficient of 4.5 × 10^− 3^ cm/h [14]. A positive displacement pipette was used to apply ~ 10 mg/cm^2^ of the compounded formulation (progesterone in either APEB or VBC) on each skin sample, and a pellet pestle was used to spread the product across the skin surface. The amount of progesterone in the skin sample was determined after 4 h.

### Quantification of permeated progesterone

The skin surface was cleaned twice with Kimwipes, and skin samples were snap-frozen by direct immersion in liquid nitrogen. The frozen samples were trimmed to the center and sectioned at 12 μm thickness using a Thermo NX50 cryostat (Thermo Scientific, San Jose, CA) and sections collected onto a standard microscope slide. Serial sections were collected of each sample for H&E staining using standard protocols [15]. Sections were dried in a desiccator for 15 min before application of the matrix.

Fiducial points were placed on the slide with the skin samples and an optical image was acquired at 4800 dpi using an Epson V600 flatbed scanner. A solution of 40 mg/mL 2,5-dihydroxybenzoic acid in 90% methanol, 0.1% trifluoroacetic acid was sprayed over the surface of the tissue section using HTX M5 Robotic Reagent Sprayer (HTX Technologies, Chapel Hill, NC). Matrix was applied over 8 passes with a solvent flow rate of 0.100 mL/min, a track speed of 1200 mm/min, a track spacing of 2 mm, a crisscross track pattern, a nitrogen gas pressure of 10 psi, a nozzle height of 40 mm, and a nozzle temperature of 75 °C.

Mass spectrometry images were collected on a Bruker timsTOF fleX QTOF mass spectrometer (Bruker Daltonics, Billerica, MA) in positive ion mode at 40 μm spatial resolution over the m/z range 100–1000. Voltages were optimized for the detection of progesterone as follows: Funnel 1 RF of 250.0 Vpp, Funnel 2 RF of 300.0 Vpp, Multipole RF of 350.0 Vpp, Collision Energy of 5.0 eV, Collision RF of 1300.0 Vpp, Quadrupole Ion Energy of 5.0 eV, Focus Pre TOF Transfer Time of 60 µs, and a Pre Pulse Storage Time of 6.0 µs. The laser was operated at 10,000 Hz and 1000 laser shots were summed up per pixel. FlexImaging 7.0 was used for data acquisition and SCiLS Lab Pro 2023c was used for data visualization and analysis.

Microscopy images of the stained sections were acquired using a Hamamatsu NanoZoomerSQ Digital Slide Scanner (Hamamatsu, Bridgewater, NJ).

### Statistical analysis

A t-test was used to determine statistical differences among mean values of progesterone contributed from the two different delivery bases. *P* values less than 0.05 were considered statistically significant. All results are expressed as mean ± SD of treatments in triplicates.

## Results

### Validation of the UPLC method

A stability-indicating assay method is critical for the determination of a BUD [16]. The UPLC method used to analyze the chemical stability of progesterone in APEB was therefore evaluated. The methods, results, and acceptance criteria for each parameter are shown in Table 1.

**Table 1:** Assay method validation parameters, corresponding methods, acceptance criteria, and results. The method was demonstrated to be linear, precise, accurate, robust, and suitable as well as stability-indicating

Parameter	Methods	Acceptance Criteria	Results
System suitability	Six replicate injections of progesterone standard solution at 100% of the target concentration were analyzed.	RSD ≤ 2.0% Tailing factor ≤ 2.0 Column Efficiency ≥ 2000	RSD = 0.224% Tailing factor = 1.22 Column Efficiency = 29,740
Linearity	Progesterone standard solutions were prepared at 5 concentrations (50%, 75%, 100%, 125%, and 150% of assay level). Each solution was injected in triplicate to generate a calibration curve.	R^2^ ≥ 0.998	Regression line: y = 20600x – 10,800 R^2^ = 0.999973
Accuracy	Spiked placebo at 50%, 100% and 150% of the assay level was prepared in triplicate. Each solution was injected and quantitated against a 5-point calibration curve.	95.0% ≤ Recovery ≤ 105.0% RSD ≤ 5.0%	Recovery at 50% = 98.3%; RSD = 0.400% Recovery at 100% = 99.2%; RSD = 0.242% Recovery at 150% = 99.7%; RSD = 0.105%
Precision (Repeatability)	A reference standard solution of progesterone at 100% assay level was prepared and analyzed 6 times.	RSD ≤ 2.0%	RSD = 0.224%
Precision (Intermediate)	Prepared triplicate spiked samples at 3 concentrations (50%, 100%, and 150% of target concentration). Samples were assayed on two different UPLC systems and on the same UPLC on two different days.	RSD ≤ 2.0%	RSD from day 1, UPLC 1 at 50% = 0.65%; at 100% = 0.53%; at 150% = 0.26% RSD from day 2, UPLC 1 at 50% = 0.40%; at 100% = 0.24%; at 150% = 0.11% RSD from day 1, UPLC 2 at 50% = 0.60%; at 100% = 0.53%; at 150% = 0.17%
Robustness	Determined with variations in column temperature, organic mobile phase content, and flow rate using spiked placebo	Resolution ≥ 1.5	Column temperature ± 2 °C Organic mobile phase ± 2% Flow rate ± 3%
Solution stability	Inter-day prepared standard solution and sample solution were analyzed.	% Difference Relative to Day 0 ≤ 2.0%	Stability (days) = 1 at 10 °C % Difference from standard = 1.0% % Difference from sample = 0.1%
Specificity	Placebo and sample were analyzed to detect degradation under stressed conditions (forced degradation studies)	No chromatogram interference 5–30% degradation in at least one stressed condition Resolution ≥ 1.5 Purity flag: No	Conditions: Thermal, oxidation, acid, and base Degradation: Yes (Thermal, acid, and base) Interference: No Resolution: ≥ 1.5 Purity flag: No

**Fig. 1:**
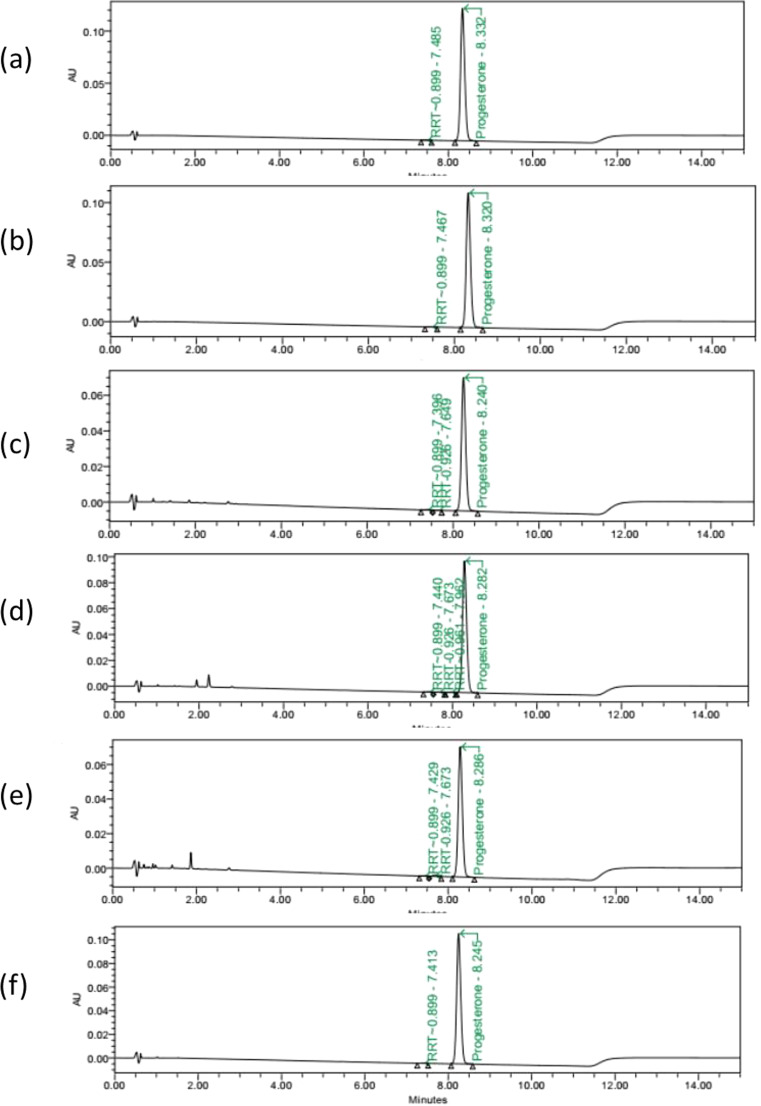
UPLC chromatograms of progesterone in APEB subjected under different conditions. Progesterone was prepared as (**a**) standard or extracted from compounded progesterone 100 mg/Gm APEB (**b**) at room temperature, after (**c**) thermal (60oC for 14 d), (**d**) acidic (0.1 N HCl for 7 d at room temperature), (**e**) basic (0.1 N NaOH for 7 d), and (**f**) oxidation (20% H2O2 for 7 d) degradation

Results from the determination of system suitability, linearity, accuracy, precision, robustness, and solution stability are all valid within the acceptable criteria. The specificity was performed to determine if the UPLC method could separate and detect progesterone from its degradation products under stressed conditions. Figure 1 shows the chemical stability of progesterone in APEB at room temperature (untreated); the elution time (8.30 min) of progesterone is almost the same as the standard progesterone (elution time = 8.32 min). Progesterone preparations exposed to heat at 60^o^C for 14 days or subjected to oxidation with 20% H_2_O_2_ for 7 days showed similar profile as the untreated preparation (Fig. 1).

Stress conditions with acid and base, however, showed minor degradation products which did not interfere with the progesterone chromatogram (Fig. 1; Table 2). All these results suggest that the UPLC method met all the necessary criteria and was considered stability-indicating for the detection and quantitation of progesterone in APEB samples.

**Table 2:** Degradation of progesterone topical gel under stressed conditions

Degradation Condition	Degradation (%)	Resolution	Purity Flag
Thermal	34.84%	8.58	No
Acid	11.13%	1.81	No
Base	34.29%	3.39	No
Oxidation	3.90%	4.85	No

### Stability of progesterone in APEB at room temperature

**Fig. 2:**
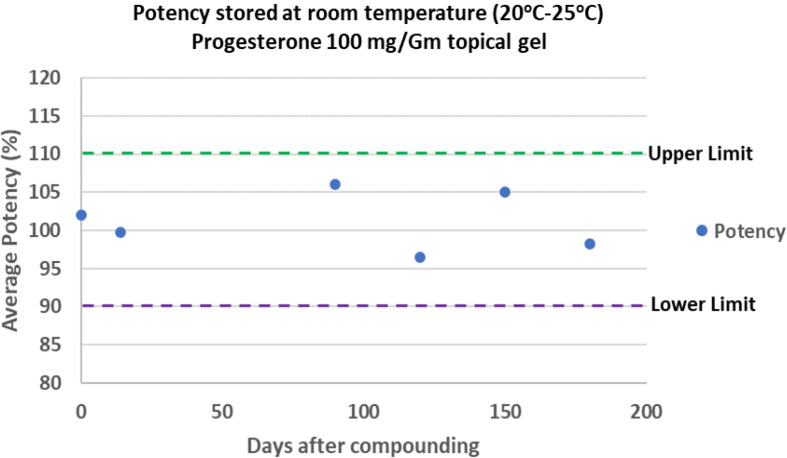
Stability of progesterone in APEB. The compounded formulation was stored at room temperature for 180 days and analyzed for the level of progesterone by UPLC. The dashed lines represent the specific range of 90.0–110.0% acceptable limits. The values are an average of four UPLC injections from two extractions

After the UPLC method was validated, it was used to determine the stability of progesterone in APEB at room temperature for 180 days. Based on the UPLC analysis, the concentration of progesterone, a reflection of its potency, ranged between 96 and 106% relative to its initial concentration (Fig. 2). The results are within the acceptable limits of 90–110%.

### Skin permeation of progesterone in APEB compared with progesterone in VBC using mass spectrometry imaging

**Fig. 3:**
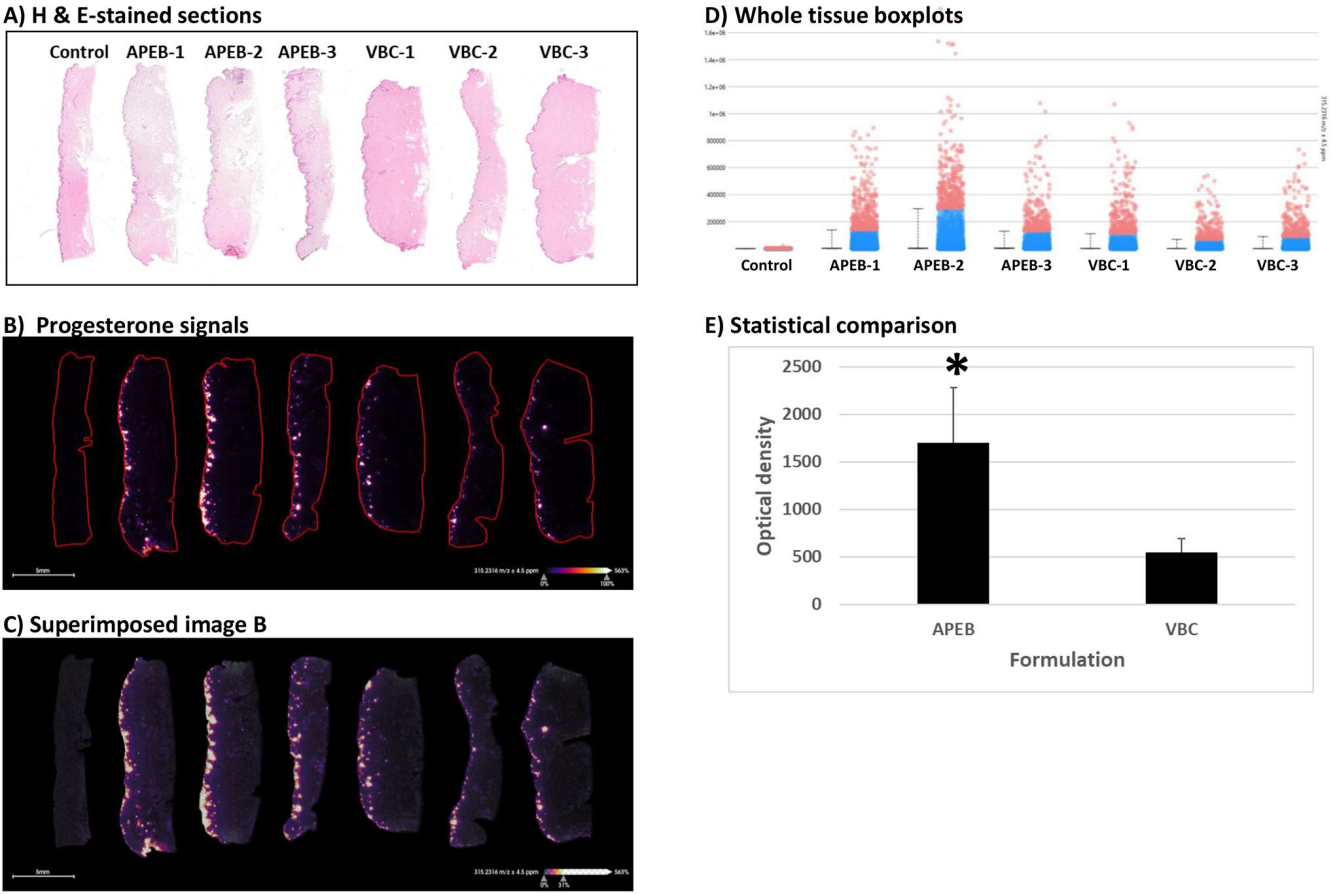
Mass spectrometric analysis of the skin permeated progesterone. Progesterone compounded in either APEB or VBC was allowed to penetrate the skin using the Franz diffusion system. Skin sections were stained (**A**), and mass spectrometry images were collected (**B**). Panel (**C**) shows the superimposed images in **B**. All spectrum signals were quantitated and presented as bar graphs in (**D**). The average optical densities of the progesterone signals were compared using the two compounding bases (**E**)

The percutaneous absorption of progesterone in APEB and VBC formulations were compared using mass spectrometry imaging. Three skin samples for each formulation were analyzed. Figure 3A, B and C show the H&E-stained tissues, progesterone signals and superimposed images. The bright signals in Fig. 3B represent progesterone which were measured quantitatively as shown in Fig. 3D. Each dot in the boxplot represents a single spectrum of progesterone from the tissue. The blue dots are spectra that are within the second and third quartile of the data and the pink dots are outside the second and third quartile of the data. Progesterone in APEB showed an average optical density of 1699 compared with progesterone in VBC which had an average optical density of 550. Statistical analysis shows a significant difference (*P* = 0.029) in the skin permeation of progesterone compounded in APEB or VBC (Fig. 3E). These results suggest that anhydrous APEB is potentially a better base to compound progesterone compared with water containing VBC.

## Discussion

The chemical composition of the compounding base is critical for the skin permeation of APIs. The methods used to quantitatively analyze the API are equally important. The present study shows that the anhydrous APEB is potentially better than the water containing VBC in facilitating the percutaneous permeation of progesterone, which was analyzed with mass spectrometry methods. A stability-indicating UPLC assay method was employed to evaluate the stability of progesterone in APEB. The stability of progesterone in APEB within 180 days at room temperature suggests a reasonable BUD for this formulation (Fig. 2). The mass spectrometry imaging method showed better penetration of progesterone through the skin when compounded in APEB, whereas VBC was relatively less superior (Fig. 3).

The better efficacy of APEB may be attributed to its chemical composition. APEB contains phosphatidylcholine and jojoba esters, which may have contributed to its permeation-enhancing property. Phosphatidylcholine, for example, was found to significantly enhance the permeation of caffeine [17] and jojoba esters, which are similar to the sebaceous lipids of the skin [18], facilitated the skin permeation of lipophilic molecules [19]. Phosphatidylcholine and jojoba esters could have made the lipid barrier of the skin more fluid, and the nonpolar nature of progesterone could have made it easier to blend with these oily components, thereby facilitating its passive permeation through the skin. The anhydrous property of APEB also provides for better solubility of the nonpolar progesterone and unfavorable conditions for growth of microorganisms, without compromising the efficacy of the skin permeation.

Although three donated skin samples were enough to show statistical differences between APEB and VBC, better statistical power and more significant comparisons could have been obtained if greater numbers were used. Moreover, additional compounding bases available in the market could have been compared with APEB in facilitating skin permeation of progesterone. The age of the donor and the organ source of the skin are important factors that may affect the skin permeation of progesterone in APEB and therefore should have been considered. In spite of these limitations, this study shows that APEB efficiently facilitates the skin permeation of progesterone and the results from this pre-clinical in vitro evaluation can be used as basis for a warranted clinical trial in using APEB as a base for compounded topical progesterone in hormone replacement therapy.

## Conclusions

APEB is a promising base for delivery of progesterone through the skin. The compounded formulation tested is stable at room temperature with a BUD of six months, an extended period that underscores the benefits and convenience of using this formulation. Moreover, mass spectrometry imaging is an effective method for the quantitative analysis of progesterone that permeated through the skin. The results from this study suggest that APEB is a reliable option for compounding pharmacists in the preparation of compounded progesterone for hormone replacement therapy.

## Data Availability

No datasets were generated or analysed during the current study.

## References

[1] Crandall CJ, Mehta JM, Manson JE (2023) Management of menopausal symptoms: A review. *JAMA*. ; 329: 405–420. 10.1001/jama.2022.24140. PMID: 36749328 10.1001/jama.2022.24140 36749328

[2] Fotherby K (1996) Bioavailability of orally administered sex steroids used in oral contraception and hormone replacement therapy. *Contraception*. ; 54:5 9–69. 10.1016/0010-7824(96)00136-9. PMID: 8842581 10.1016/0010-7824(96)00136-9 8842581

[3] Kuhl H (2005) Pharmacology of estrogens and progestogens: influence of different routes of administration. *Climacteric*. ; 8(Suppl 1): 3–63. doi: 10.1080/13697130500148875. PMID: 16112947 10.1080/13697130500148875 16112947

[4] Salem HF, Kharshoum RM, Abou-Taleb HA, AbouTaleb HA, AbouElhassan KM (2019). Progesterone-loaded nanosized transethosomes for vaginal permeation enhancement: formulation, statistical optimization, and clinical evaluation in anovulatory polycystic ovary syndrome. J Liposome Res.

[5] Goletiani NV, Keith DR, Gorsky SJ (2007). Progesterone: review of safety for clinicalstudies. Exp Clin Psychopharmacol.

[6] Patil N, Maheshwari R, Wairkar S (2023). Advances in progesterone delivery systems: still work in progress?. Int J Pharm.

[7] Elgindy NA, Mehanna MM, Mohyeldin SM (2016). Self-assembled nano-architecture liquid crystalline particles as a promising carrier for progesterone transdermal delivery. Int J Pharm.

[8] Song G, Banov D, Song H, Liu Y, IP K, Bassani AS, Valdez BC (2022) Evaluation of an anhydrous permeation-enhancing vehicle for percutaneous absorption of hormones. *AAPS PharmSciTech*. ; 23(6):198. 10.1208/s12249-022-02352-3.PMID: 35854200 10.1208/s12249-022-02352-3 35854200

[9] International Council for Harmonisation of Technical Requirements for Pharmaceuticals for Human Use (2005) ‘Validation of Analytical Procedures: Text and Methodology Q2(R1)’. Quality Guidelines. http://www.ich.org/fileadmin/Public_Web_Site/ICH_Products/Guidelines/Quality/Q2_R1/Step4/Q2_R1__Guideline.pdf

[10] The United States Pharmacopeial Convention (2018). General chapters / <1225 > validation of compendial procedures. USP 40 – NF 35 S2.

[11] Ip K, Song G, Banov D, Bassani AS, Valdez BC (2020). In vitro evaluation of Naltrexone HCl 1% topical cream in XemaTop™ for psoriasis. Arch Dermatol Res.

[12] Bassani AS, Banov D, Carvalho M (2017). Evaluation of the in vitro human skin percutaneous absorption of progesterone in Versabase® using the Franz skin finite dose model. J Women’s Health Care.

[13] Davies DJ, Ward RJ, Heylings JR (2004). Multi-species assessment of electrical resistance as a skin integrity marker for in vitro percutaneous absorption studies. Toxicol Vitro.

[14] Median VM, Roper CS (2008). Inter- and intra-individual variability in human skin barrier function: a large scale retrospective study. Toxicol Vitro.

[15] Feldman AT, Wolfe D (2014). Tissue processing and hematoxylin and eosin staining. Methods Mol Biol.

[16] Kupiec TC, Skinner R, Lanier L (2008). Stability versus potency testing: the madness is in the method. Int J Pharm Compd.

[17] Kim C, Shim J, Han S, Chang I (2002). The skin-permeation-enhancing effect of phosphatidylcholine: caffeine as a model active ingredient. J Cosmet Sci.

[18] Wertz PW (2009) Human synthetic sebum formulation and stability under conditions of use and storage. *Int J Cosmet Sci*. ; 31: 21–25. 10.1111/j.1468-2494.2008.00468.x. PMID: 19134124 10.1111/j.1468-2494.2008.00468.x 19134124

[19] Gad HA, Roberts A, Hamzi SH, Gad HA, Touiss I, Altyar AE (2021). Jojoba oil: an updated comprehensive review on chemistry, pharmaceutical uses, and toxicity. Polym (Basel).

